# Negative Life Events, Social Ties, and Depressive Symptoms for Older Adults in China

**DOI:** 10.1093/geroni/igaa057.1931

**Published:** 2020-12-16

**Authors:** Hangqing Ruan, Feinian Chen

**Affiliations:** 1 University of Maryland, Hyattsville, Maryland, United States; 2 University of Maryland, College Park, College Park, Maryland, United States

## Abstract

Negative life events are considered important risk factors of depression among older adults. An overwhelming amount of literature suggests that individuals with the most supportive social relations tend to make a better recovery from stressful life events. As for which types of ties matter the most, whether being family, relatives, friends or the broader community, existing literature is much less consistent and has documented varying effects across different contexts. This study is set in China, which traditionally relies on family systems and filial obligations for old-age support. Using two waves of data from China Longitudinal Aging Social Survey, we examine the protective effect of different types of social relations on depressive symptoms, including those who are living in the household, children who live close by or far away, as well as their ties with family, relatives, and friends.

